# The Burden of JAK2V617F Mutated Allele in Turkish Patients With Myeloproliferative Neoplasms

**DOI:** 10.14740/jocmr2047w

**Published:** 2014-12-29

**Authors:** Ipek Yonal-Hindilerden, Aynur Daglar-Aday, Basak Akadam-Teker, Ceylan Yilmaz, Meliha Nalcaci, Akif Selim Yavuz, Deniz Sargin

**Affiliations:** a Division of Hematology, Department of Internal Medicine, Istanbul University Istanbul Medical Faculty, Fatih, Istanbul, Turkey

**Keywords:** Philadelphia-negative myeloproliferative neoplasms, Essential thrombocythemia, Primary myelofibrosis, JAK2V617F allele burden, JAK2V617F allele status

## Abstract

**Background:**

Studies regarding the impact of JAK2V617F allele burden on phenotypic properties and clinical course in Philadelphia-negative myeloproliferative neoplasms (Ph-negative MPNs) have reported variable results. We aimed to analyze the association of mutated JAK2V617F allele burden with laboratory characteristics and clinical phenotype in Turkish patients (107 essential thrombocythemia (ET) and 77 primary myelofibrosis (PMF)).

**Methods:**

Peripheral blood samples of 184 patients with Ph-negative MPNs were analyzed for JAK2V617F allele status and burden. JAK2 MutaScreen assay (Ipsogen, Luminy Biotech, Marseille, France) was used to detect the JAK2V617F status and quantitative JAK2V617F allele burdens in genomic DNA using TaqMan allelic discrimination.

**Results:**

Frequency of JAK2V617F-positive patients with high mutation load (allele burden > 50%) was higher in PMF compared to ET (23.4% and 4.7%, respectively; P = 0.001). We found significant association between ET patients with high JAK2V617F allele burden and lower hemoglobin (Hgb) and hematocrit (Hct), higher LDH levels and more prevalent massive splenomegaly (P = 0.001, P = 0.001, P = 0.012 and P = 0.015, respectively). ET patients with high mutation load displayed higher prevalence of bleeding compared to low mutation load and wild-type mutational status (P = 0.003). Rate of DVT was significantly higher in ET patients with mutant allele burden in upper half compared to lower half and wild-type (P = 0.029). We observed significant association between PMF patients with high JAK2V617F allele burden and higher Hgb, Hct levels and leukocyte counts (P = 0.003, P = 0.021 and P = 0.001, respectively).

**Conclusions:**

Our study demonstrated JAK2V617F allele burden correlates with clinical features in ET and PMF. We conclude quantification of JAK2V617F mutation contributes to the workup of Ph-negative MPNs.

## Introduction

Philadelphia-negative (Ph-negative) myeloproliferative neoplasms (MPNs) are clonal hematopoietic stem cell disorders that comprise a group of conditions including polycythemia vera (PV), essential thrombocythemia (ET) and primary myelofibrosis (PMF) [1]. All three disorders are characterized by unregulated proliferation that leads to the accumulation of mature-appearing blood cells within the blood stream. The discovery of a point mutation in the Janus kinase 2 (JAK2) gene provided a molecular basis for the pathophysiology of Ph-negative MPNs [2]. The mutation is harbored by more than 90% in PV and about 60% in ET or PMF. These three disorders differ in phenotypic properties. PV and ET are mainly characterized by microcirculatory disturbances and a tendency to develop thrombohemorrhagic complications [3]. PMF has a more severe course with a median survival of about 5 years and death is mainly due to leukemic transformation, accounting for 20% of the patients [4]. The JAK2V617F mutational status is now included as a major criterion to rule out reactive myeloproliferation in the current World Health Organization (WHO) classification of myeloid neoplasms [5]. Therefore, it must be considered in the initial diagnostic workup in patients with MPN. Several studies have reported different JAK2V617F allele burden in these entities. It has been noticed that the highest and lowest values were expressed by patients with post-PV/post-ET myelofibrosis and ET, respectively and the intermediate burden was detected in PV or PMF patients [6]. Differences in mutant allele burden may be a potential explanation for the differences in phenotypic properties among these disorders. Researchers in various studies have reported variable results regarding the impact of JAK2V617F allele burden on phenotypic properties and clinical course in Ph-negative MPNs [6-12]. Several studies showed that high JAK2V617F mutation load correlated with a high frequency of thrombotic events and predicted myelofibrotic transformation in PV and ET while other studies indicated that in PMF, low JAK2V617F allele load was associated with poor survival [7, 8, 13-18]. We sought to analyze the association of mutated JAK2V617F allele burden with laboratory characteristics and clinical phenotype in a total of 184 Turkish patients (107 ET and 77 PMF).

## Materials and Methods

### Patients

Our study group involved a total of 184 consecutive ET and PMF patients diagnosed between May 1995 and July 2013. Peripheral blood samples were collected at the outpatient clinic of Hematology Department at Istanbul University Istanbul Medical Faculty. The clinical diagnosis was based on the 2008 WHO criteria [5]. The subtypes of 184 Ph-negative MPNs were as follows: ET (n = 107) and PMF (n = 77). This study was approved by local ethics committee and performed according to the principles of the Declaration of Helsinki. Informed consent was obtained from all participants. Data obtained at study entry involved demographics, diagnostic features and clinical complications. Medical histories of red blood cell transfusion, phlebotomy, splenectomy and medications were recorded. Risk factors for cardiovascular diseases were questinoned. Spleen longitudinal diameter ≥ 130 mm up to 160 mm and ≥ 160 mm on ultrasound were regarded as mild and massive splenomegaly, respectively. Risk categories of PMF patients were determined according to Dynamic International Prognostic Scoring System (DIPSS)-plus [19]. Karyotypes were allocated according to International System for Human Cytogenetic Nomenclature (ISCN) guidelines [20]. Unfavorable karyotypes were as follows: complex karyotype or sole or two abnormalities that include +8, -7/7q-, i(17q), inv(3), -5/5q-, 12p-, or 11q23 rearrangement [21]. Cytogenetic abnormalities other than the above-mentioned categories were defined as favorable karyotype abnormalities. Patients with no cytogenetic abnormalities were categorized to have normal karyotype.

### Genomic analysis

JAK2V617F allele burden was assessed in 107 ET (58 females and 49 males) and 77 PMF (43 females and 34 males) patients at the Molecular Hematology Laboratory of Istanbul University between January 2011 and July 2013. Genomic DNA was extracted from peripheral blood granulocytes using the high pure polymerase chain reaction (PCR) template preparation kit (Roche Diagnostic, Mannheim, Germany). DNA concentration was measured using a Nano-Drop-2000 spectrophotometer (Thermo Scientific, Wilmington, DE, USA).

### Quantification of JAK2V617F allele burden

JAK2 MutaScreen assay (Ipsogen, Luminy Biotech, Marseille, France) was used to detect the JAK2V617F status and quantitative JAK2V617F allele burdens in genomic DNA using TaqMan allelic discrimination [22, 23]. The assay is based on the simultaneous use of two specific TaqMan probes (Applied Biosystems) and the measurement of the respective fluorescence of the two alleles (FAM for V617F and VIC for wild-type) to differentiate the amplification of each allele. PCR amplifications were done in a total volume of 25 μL PCR mix containing 12.5 μL of TaqMan Universal Master Mix, 5 μL of nuclease-free PCR grade water, 2.5 μL of primers and probes mix and 50 ng/μL of DNA template. Reactions were performed using the following PCR conditions: initial denaturation step of 95 °C for 15 min, followed by 50 cycles of amplification consisting of denaturation at 95 °C for 15 s, annealing at 60 °C for 1 min, and extension at 60 °C for 20 s. Quantitation of mutant and wild-type alleles was performed by using a Rotor-Gene 3000 real-time PCR instrument (Corbett Research, Sydney, Australia). Mutant allele burden was reported as the percentage of total JAK2 represented by JAK2V617F (that is JAK2V617F + JAK2 wild type). The JAK2V617F allele burden was estimated by six-scaled standards of the mutant allele (2%, 5%, 12.5%, 31%, 50%, and 78%). JAK2V617F mutant allele burden equal to or less than 50% and greater than 50% were named as low and high JAK2V617F allele burden, respectively.

### Statistical analyses

The JAK2V617F mutated allele burden was considered as an ordered categorical variable based on the following groups: 0% (JAK2 wild-type), equal to or less than 50%, and greater than 50%.

All statistical calculations were performed using the SPSS version 16 (Prentice Hall, Upper Saddle River, NJ, USA). Numerical variables were summarized by mean (SD). The Chi-squared statistics were used to compare categorical variables among the different patient groups that had been categorized according to the mutated allele burden. Comparison between categorical and continuous variables was performed by either the Mann-Whitney U test (two groups) or Kruskal-Wallis test (more than two groups). A two-tailed P value of less than 0.05 was considered to indicate statistical significance. The analysis of correlation between JAK2V617F allele burden and clinical and laboratory variables was performed according to Spearman’s rank correlation test.

## Results

**Figure 1:**
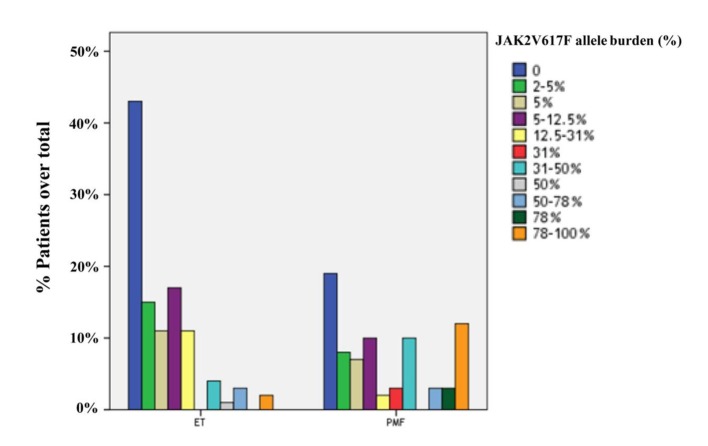
Distribution of ET (n = 107) and PMF (n = 77) patients in quartiles according to the JAK2V617F allele burden. The prevalences of JAK2 wild-type in ET and PMF patients were 40.2% and 24.7%, respectively. The frequency of JAK2V617F-positive patients with mutant allele burden in upper half (allele burden > 50%) was higher in PMF compared to ET (23.4% and 4.7%, respectively; P = 0.001).

JAK2V617F was detected in 122 of 184 cases (66.3%), including 58 cases with PMF (75.3%) and 64 cases with ET (59.8%). The frequency of JAK2V617F mutation was significantly higher in PMF than ET (P = 0.028). Furtheron, the prevalence of JAK2V617F-positive patients with high allele burden was greater in PMF compared to ET (23.4% and 4.7%, respectively; P = 0.001). The distributions of quartile values according to JAK2V617F allele burden in ET and PMF patients are shown in Figure 1.

### Comparison of ET patients according to the JAK2V617F allele burden data

The mean age of ET patients (54.2% females) was 56.3 years (SD 14.5). Of 107 ET patients, 64 (59.8%) harbored JAK2V617F mutation. ET patients with JAK2V617F allele burden data were divided into three groups: JAK2V617F mutation-negative (n = 43), JAK2V617F-positive with low mutation load (allele burden ≤ 50%, n = 59) and high mutation load (allele burden > 50%, n = 5).

Clinical and laboratory characteristics of ET patients according to JAK2V617F allele burden data were outlined in Tables 1 and 2.

**Table 1:** Clinical and Laboratory Features of Patients With ET According to JAK2V617F Allele Burden Data

ET	JAK2 wild-type, mean (SD)	Low JAK2 allele burden (≤ 50%), mean (SD)	High JAK2 allele burden (> 50%), mean (SD)	P value
Number of patients	43	59	5	-
Age at diagnosis (years)	51.7 (15.7)	49.4 (14.6)	53.2 (19)	0.732
Females (%)	20 (46.5%)	34 (57.6%)	4 (80%)	0.267
Leukocyte at diagnosis (mm^3^)	9.593 (3.434)	9.853 (3.663)	14.240 (7.279)	0.263
Hgb at diagnosis (g/dL)	12.4 (1.9)	13.8 (1.7)	11.1 (0.9)	0.001
Hct at diagnosis (%)	36.8 (5.21)	41.2 (5.15)	34.8 (4.76)	0.001
Platelet count at diagnosis (mm^3^)	1.055.116 (495.928)	881.238 (323.317)	798.600 (312.966)	0.123
LDH at diagnosis (U/L)	462 (159.7)	430.7 (125.2)	718 (178.6)	0.012
Spleen size at diagnosis (mm)	132.07 (23.86)	138.3 (31.4)	181 (74)	0.123
Follow-up duration (months)	70.1 (56.9)	69.76 (62.9)	69.2 (80.7)	0.887

**Table 2:** Clinical and Laboratory Features of Patients With ET According to JAK2V617F Allele Burden Data (Continued)

ET	JAK2 wild-type, n (%)	Low JAK2 allele burden (≤ 50%), n (%)	High JAK2 allele burden (> 50%), n (%)	P value
Number of patients	43	59	5	-
Risk factors for cardiovascular diseases	27 (62.8%)	44 (74.6%)	5 (100%)	0.148
Splenomegaly group	43 (100%)	59 (100%)	5 (100%)	0.015
No splenomegaly	33 (76.8%)	33 (56%)	1 (20%)	-
Mild splenomegaly	5 (11.6%)	16 (27.1%)	1 (20%)	-
Massive splenomegaly	5 (11.6%)	10 (16.9%)	3 (60%)	-
Bleeding	3 (7%)	7 (11.9%)	3 (60%)	0.003
Need for red blood cell transfusion	4 (9.3%)	0	1 (20%)	0.022
Need for phlebotomy	1 (2.3%)	4 (6.8%)	0	0.5
Hydroxyurea	35 (81.4%)	52 (88.1%)	5 (100%)	0.408
History of splenectomy	1 (2.3%)	1 (1.7%)	0	0.926
ASA	41 (95.3%)	48 (81.4%)	4 (80%)	0.105
Thrombosis	15 (34.9%)	24 (40.7%)	2 (40%)	0.835
Thrombosis group	43 (100%)	59 (100%)	5 (100%)	0.377
No thrombosis	28 (65.1%)	35 (59.4%)	3 (60%)	-
Arterial	10 (23.3%)	11 (18.6%)	0	-
Venous	4 (9.3%)	12 (20.3%)	2 (40%)	-
Arterial and venous	1 (2.3%)	1 (1.7%)	0	-
Death	2 (4.7%)	2 (3.4%)	1 (20%)	0.24

No significant differences were observed in gender and age at diagnosis between the three groups. In the comparison across the three groups, lower hemoglobin (Hgb) and hematocrit (Hct) levels at diagnosis were significantly associated with high mutation load (P = 0.001, each). ET patients with high mutation load had higher LDH levels at diagnosis compared to the other two groups (P = 0.012). Leukocyte and platelet counts did not differ in any of the categories.

There was no significant difference in mean spleen size between the three groups, although massive splenomegaly was significantly more prevalent in ET patients with JAK2V617F allele burden greater than 50% (P = 0.123 and P = 0.015, respectively). No significant differences were observed in the rate of phlebotomy and the presence of risk factors for cardiovascular diseases between the three groups.

The percentage of bleeding events in patients with high mutation load was as high as 60% compared to 11.9% in low mutation load and 7% in wild-type JAK2 (P = 0.003). In line with this observations, the need for red blood cell transfusion was significantly higher in ET patients with JAK2V617F allele burden greater than 50% (P = 0.022).

The prevalence of major thrombotic events and arterial thrombosis did not significantly differ among the three groups (P = 0.835 and P = 0.428, respectively). In addition, no significant difference was observed in the rate of venous thrombosis between the three categories (P = 0.142). As regards to the localization of venous thrombosis among ET patients, 20% of the ET patients with high JAK2V617F allele burden experienced deep vein thrombosis (DVT) whereas the frequency of DVT was 2.3% in patients with wild-type JAK2. No DVT developed in ET patients with low allele burden (P = 0.029). Consequently, the rate of DVT was higher in ET patients with high mutation load compared to the other two groups (P = 0.029).

There was no significant difference in the prevalence of hydroxyurea use, acetylsalicylic acid (ASA) use and the rate of splenectomy between the three groups. In addition, no significant differences were observed in the use of other medical treatments in any of the categories (P > 0.05). Durations of follow-up in patients with high mutation load, low mutation load and wild-type mutational status were 69.2 months (SD 80.7), 69.7 months (SD 62.9) and 70.1 months (SD 56.9), respectively (P = 0.887). During follow-up, one of five (20%) patients with high allele burden deceased. The rates of death in wild-type mutational status and low allele burden were 4.7% and 3.4%, respectively (P = 0.24).

ET patients were further analyzed for the correlation of JAK2V617F allele burden with clinical and laboratory variables using the Spearman rank correlation test. In ET, JAK2V617F allele burden correlated with the leukocyte count, bleeding complications and venous thrombosis (r = 0.238, r = 0.213 and r = 0.201, respectively). In addition, we observed a weak positive correlation between Hct level and ET patients with low mutation load (r = 0.276). In this population, JAK2V617F allele burden did not correlate with platelet count, LDH level, spleen size, total thrombotic events and arterial thrombosis (r < 0.2).

### Comparison of PMF patients according to the JAK2V617F allele burden data

A total of 77 PMF patients (55.8% female; mean age 60.8 (SD 14.5)) were included. JAK2V617F mutation was detected in 58 PMF patients (75.3%). PMF patients with JAK2V617F allele burden data were divided into three groups: JAK2V617F mutation-negative (n = 19), JAK2V617F-positive with mutant allele burden in low mutation load (allele burden ≤ 50%, n = 40) and high mutation load (allele burden > 50%, n = 18).

Clinical and laboratory parameters of PMF patients divided according to JAK2V617F allele burden data were summarized in Tables 3 and 4.

**Table 3:** Clinical and Laboratory Parameters of PMF Patients Divided by JAK2V617F Allele Burden Data

PMF	JAK2 wild-type, mean (SD)	Low JAK2 allele burden (≤ 50%), mean (SD)	High JAK2 allele burden (> 50%), mean (SD)	P value
Number of patients	19	40	18	-
Age at diagnosis (years)	52.8 (16)	56.7 (15.2)	61.2 (9.5)	0.192
Females (%)	16 (84.2%)	19 (47.5%)	8 (44.4%)	0.011
Leukocyte at diagnosis (mm^3^)	9.726 (7.875)	11.597 (9.018)	26.216 (19.374)	0.001
Hgb at diagnosis (g/dL)	9.4 (1.3)	10.7 (2.3)	11.76 (1.9)	0.003
Hct at diagnosis (%)	29.4 (4.81)	31.8 (7.71)	35.5 (6.1)	0.021
Platelet count at diagnosis (mm^3^)	464.526 (396.324)	377.302 (341.495)	526.777 (367.513)	0.184
LDH at diagnosis (U/L)	782 (364.5)	808 (350)	921 (508.7)	0.855
Spleen size at diagnosis (mm)	183.7 (37.3)	196.9 (42.8)	213.8 (46.3)	0.22
Follow-up duration (months)	56.6 (48.7)	42.8 (52.1)	40.1 (33.9)	0.359

**Table 4:** Clinical and Laboratory Parameters of PMF Patients Divided by JAK2V617F Allele Burden Data (Continued)

PMF	JAK2 wild-type, n (%)	Low JAK2 allele burden (≤ 50%), n (%)	High JAK2 allele burden (> 50%), n (%)	P value
Number of patients	19	40	18	-
Risk factors for cardiovascular diseases	10 (52.6%)	25 (62.5%)	11 (61.1%)	0.763
Splenomegaly group	19 (100%)	40 (100%)	18 (100%)	0.115
No splenomegaly	1 (5.3%)	0	0	-
Mild splenomegaly	6 (31.6%)	10 (25%)	1 (5.6%)	-
Massive splenomegaly	12 (63.2%)	30 (75%)	17 (94.4%)	-
Bleeding	1 (5.3%)	11 (27.5%)	3 (16.7%)	0.124
Need for red blood cell transfusion	3 (15.8%)	16 (40%)	2 (11.1%)	0.032
Need for phlebotomy	0	0	1 (5.6%)	0.19
Hydroxyurea	18 (94.7%)	36 (90%)	18 (100%)	0.349
History of splenectomy	1 (5.3%)	3 (7.5%)	0	0.492
ASA	13 (68.4%)	20 (50%)	14 (77.8%)	0.1
AHSCT	1 (5.3%)	2 (5%)	0	0.62
Karyotype	19 (100%)	40 (100%)	18 (100%)	0.572
Normal	18 (94.7%)	34 (85%)	15 (83.3%)	-
Favorable	0	5 (12.5%)	2 (11.1%)	-
Unfavorable	1 (5.3%)	1 (2.5%)	1 (5.6%)	-
DIPSS-plus	19 (100%)	40 (100%)	18 (100%)	0.076
Low risk	4 (21%)	7 (17.5%)	4 (22.2%)	-
Intermediate-1	5 (26.3%)	13 (32.5%)	9 (50%)	-
Intermediate-2	10 (52.6%)	12 (30%)	5 (27.8%)	-
High risk	0	8 (20%)	0	-
Thrombosis	3 (15.8%)	6 (15%)	2 (11.1%)	0.905
Thrombosis group	19 (100%)	40 (100%)	18 (100%)	0.483
No thrombosis	16 (84.2%)	34 (85%)	16 (88.9%)	-
Arterial	3 (15.8%)	2 (5%)	2 (11.1%)	-
Venous	0	3 (7.5%)	0	-
Arterial and venous	0	1 (2.5%)	0	-
Leukemic transformation	1 (5.3%)	2 (5%)	1 (5.6%)	0.996
Death	3 (15.8%)	8 (20%)	3 (16.7%)	0.909

There were no significant differences in age at diagnosis between these subcohorts. PMF patients with wild-type JAK2 had a higher rate of females compared to the other two groups (P = 0.011). In the comparison across the three groups, the high mutation load was significantly associated with higher Hgb and Hct levels and leukocyte counts at diagnosis (P = 0.003, P = 0.021 and P = 0.001, respectively). The platelet count and LDH level were similar between the three groups.

The rate of phlebotomy, presence of risk factors for cardiovascular diseases, mean spleen size and degree of splenomegaly did not differ in any of the categories. The need for red blood cell transfusion in patients with low mutation load was as high as 40% compared to 15.8% wild-type mutational status and 11.1% in high mutation load (P = 0.032). Patients with low mutation load showed a higher yet not statistically significant prevalence of bleeding events compared to the other two groups. The prevalence of total thrombotic events, arterial thrombosis and venous thrombosis did not significantly differ among the three groups (P = 0.905, P = 0.322 and P = 0.142, respectively).

There was no significant difference in the prevalence of hydroxyurea use, ASA use, the rate of allogeneic hematopoietic stem cell transplantation (AHSCT) and history of splenectomy between the three groups. In addition, no significant differences were observed in the use of other medical treatments in any of the categories (P > 0.05).

Distribution of karyotype categories was similar between the groups. PMF patients with low mutation load showed a trend towards higher rate of DIPSS-plus high risk categories compared to PMF patients with high mutation load and wild-type mutational status (20%, 0 and 0, respectively; P = 0.076).

Durations of follow-up in patients with high mutation load, low mutation load and wild-type mutational status were 40.1 months (SD 33.9), 42.8 months (SD 52.1) and 56.6 months (SD 48.7), respectively (P = 0.359). At the end of data collection period, eight of 40 (20%) patients with low allele burden succumbed to the disease while the rates of death in high allele burden and wild-type mutational status were 16.7% and 15.8%, respectively (P = 0.909). During follow-up, the rate of leukemic transformation was similar between the three categories.

In PMF patients, the load of JAK2 mutant allele correlated with Hct level (r = 0.318). Also, we observed a mild positive correlation between leukocyte count and PMF patients with high allele burden (r = 0.396). JAK2V617F allele burden did not correlate with platelet count, LDH level, spleen size, total thrombotic events, arterial thrombosis, venous thrombosis and bleeding complications (r < 0.2).

## Discussion

JAK2V617F mutation was identified in around 95% of patients with PV, 65% of patients with PMF and 55% of ET [24-26]. Another study reported the frequency of JAK2V617F mutation in the range of 50-70% among ET patients [27]. The literature suggested that a sensitivity of 1-5% for the JAK2V617F quantification assays was clinically appropriate and sufficient [28, 29]. Using JAK2 MutaScreen assay (assay sensitivity of 2%) to detect JAK2V617F, the presence of JAK2V617F mutation in our PMF patients was found to be significantly higher as compared to ET patients (75.3% and 59.8%, respectively). In our study, the prevalence of JAK2V617F mutation in ET patients was consistent with previous data whereas in PMF patients, we observed a higher incidence of JAK2V617F mutation than previously reported. This difference is probably due to the characteristics of our study population, which includes MPN patients of a reference center, most of which have already documented JAK2V617F mutation.

Several studies have shown that homozygosity for the JAK2V617F mutation (mutant allele burden greater than 50%) is displayed by 25-30% of PMF patients as opposed to 2-4% of ET patients [2, 4, 7, 17]. In another series of PMF patients, homozygosity for the JAK2V617F mutation was reported as 27.6% [11]. In the study by Antonioli et al high JAK2V617F allele burden was found in 5% of ET patients [6]. In summary, the findings in all the above studies indicated that PMF patients have a greater prevalence of high JAK2V617F mutation load compared to ET patients. Confirming previous observations, we found that the frequency of JAK2V617F-positive patients with high mutation load was greater in PMF compared to ET patients (23.4% and 4.7%, respectively).

Several previous studies investigated the clinical and laboratory parameters of ET patients divided according to JAK2V617F allele burden [6-8, 17, 18, 30-32]. In the study by Pich et al, JAK2V617F-positive ET patients were divided as follows: high JAK2V617F allele load (mutation load > 12.5%) and low allele load (mutation load ≤ 12.5%) [30]. According to this study, no differences in age, leukocyte count and Hct level were observed between the two groups whereas ET patients with high mutation load had lower Hgb level and platelet count, higher LDH level, larger spleen and more prevalent venous and arterial thrombosis as opposed to patients with low mutation load [30]. In another study, mutant allele burden in ET patients correlated with platelet and leukocyte counts, palpable splenomegaly and venous thrombosis after diagnosis [8]. Conversely, in the same study, mutant allele burden in ET did not significantly correlate with gender, arterial thrombosis or major hemorrhage [8]. Antonioli et al reported that greater JAK2V617F allele burden in ET patients correlated with older age, larger spleen, microvessel disease, arterial thrombosis but not with hemorragic events [6]. In the study by Vannucchi et al homozygous ET patients were older, displayed a higher leukocyte count and Hct value at diagnosis, presented with larger spleen volume and portended a significantly higher risk of cardiovascular events than in wild-type or in heterozygous patients whereas no differences in gender and platelet count were observed between the above-mentioned groups [7]. In the aforementioned study, thrombotic events were more prevalent in homozygous patients compared to the other two groups [7]. Vannucchi et al reported that 21.4% of homozygous ET patients presented with DVT at diagnosis [7]. In the study by Vannucchi et al, the frequency of major hemorrhages that developed during follow-up was higher in homozygous ET patients than in wild-type or heterozygous ET patients [7]. In another series of 49 ET patients, the occurrence of thrombosis at diagnosis was more frequent in patients with allele burden > 50% whereas mutant allele burden did not influence hemorrhagic episodes [18]. Dupont et al demonstrated that higher levels of mutant JAK2V617F in the ET group are associated with higher platelet and leukocyte counts [32]. On the other hand, in the same study, JAK2V617F allele burden did not correlate with age, Hgb level and Hct level [32]. In summary, according to the literature, data on ET regarding the impact of JAK2V617F mutation load on clinical and laboratory parameters are conflicting.

In our study, ET patients were divided into three groups based on JAK2V617F allele burden: JAK2V617F mutation-negative (n = 43), JAK2V617F-positive with low allele burden (allele burden ≤ 50%, n = 59) and high allele burden (allele burden > 50%, n = 5). In the current study, ET patients with high mutation load had lower Hgb and higher LDH levels in line with the report by Pich et al [30]. In addition, we observed lower Hct levels in ET patients with high mutation load while no differences were found between the groups regarding age, gender, platelet counts, presence of risk factors for cardiovascular diseases and the rate of phlebotomy. Using Kruskal-Wallis test, we observed statistically insignificant higher leukocyte counts in ET patients with high mutation load than those with low mutation load and wild-type mutational status (mean 14.240/mm^3^ (SD 7.279), 9.853/mm^3^ (SD 3.663) and 9.593/mm^3^ (SD 3.434), respectively). On the other hand, using the Spearman rank correlation test, we found a weak positive correlation between leukocyte count and ET patients with high mutation load in accordance with some previous reports [7, 8, 32]. There were no significant differences in mean spleen size and the rate of splenectomy between our study groups whereas a higher incidence of massive splenomegaly was observed in our ET patients with high mutation load.

At variance with some previous studies, mutant allele burdens in our ET group did not correlate with total thrombotic events and arterial thrombosis. Using Kruskal-Wallis test, no significant difference was observed in the prevalence of venous thrombosis between our study groups. On the other hand, using the Spearman rank correlation test, we found a weak positive correlation between venous thrombosis and ET patients with high mutation load. When the types of venous thrombosis were considered separately, the rate of DVT was found significantly higher in ET patients with high mutation load compared to low mutation load and wild-type patients (20%, 0 and 2.3%, respectively). Consequently, consistent with the report of Vannucchi et al, we observed increased rate of DVT in our ET patients with high mutation load [7]. As opposed to some previous reports yet consistent with the findings of Vannucchi et al, we observed higher prevalence of bleeding events in ET patients with high mutation load than in patients with low mutation load and wild-type mutation [6-8, 18]. Consequently, the need for red blood cell transfusion was significantly higher in our ET patients with JAK2V617F allele burden greater than 50%.

In the study of Vannucchi et al, ET patients homozygous for JAK2V617F mutation displayed a higher incidence of cytoreductive therapy requirement almost at the level of statistical significance [7]. In contrast, Antonioli et al reported that the mutant JAK2V617F allele burden in ET had no impact on the need for cytoreductive treatment [6]. Confirming the aforementioned study, in our ET patients, the prevalence of hydroxyurea use and other medical treatments did not differ in any of the categories.

Kittur et al reported that increased mutant allele burden did not significantly affect survival in ET patients [8]. Confirming this observation, the rate of death in our ET patients with high allele burden was higher, yet showing no statistical significance compared to patients with low allele burden and wild-type mutational status (20%, 3.4% and 4.7%, respectively).

As a whole, results obtained in ET patients are ambiguous. Vannucchi et al reported that more than 90% of ET patients belong to the low allele burden (allele burden ≤ 50%); this strict distribution of mutational load probably accounts for the controversies in the phenotypic characteristics of ET patients when classified according to the allele burden [17].

Finally, in aggreement with the study by Pich et al, we have documented that ET patients with high JAK2V617F allele burden (allele burden > 50%) had a more severe disease with lower Hgb and Hct levels, higher LDH levels and more prevalent massive splenomegaly [30]. Furthermore, our ET patients with high mutation load displayed increased rate of DVT and higher prevalence of bleeding events in line with the results of Vannucchi et al [7].

A limited number of studies have investigated the association of the clinical and laboratory parameters with JAK2V617F mutation load in PMF patients [11, 12, 31, 33, 34]. Barosi et al reported that PMF patients with high mutation load (allele burden > 50%) displayed higher leukocyte count, larger splenomegaly, longer disease duration and were more likely to require cytoreductive therapy or splenectomy [11]. On the other hand, in the same study, mutant allele burden in PMF did not significantly correlate with age, gender and LDH levels [11]. In the study by Tefferi et al, PMF patients with mutant allele burden data were divided into four groups: JAK2V617F wild-type mutational status and JAK2V617F-positive with mutant allele burden in the lower quartile (allele burden: 1-20%), middle quartile (allele burden: 21-55%) or upper quartile (allele burden: 56-74%) ranges [12]. This study revealed significant association between lower quartile allele burden and older age and higher leukocyte count in patients with upper quartile allele burden [12]. Conversely, in the same study, gender, Hgb level, platelet count, median follow-up, rate of cytoreductive therapy and any other treatments, rate of splenectomy, frequency of thrombosis, and bleeding did not differ between the four groups [12]. Guglielmelli et al reported that PMF patients in the upper quartile had higher Hgb level, leukocyte count, longer disease duration and higher frequency of large splenomegaly compared to those in lower quartiles [33]. The aforementioned study revealed no statistical significance in age, gender, platelet count and LDH level in relation to JAK2V617F allele burden [33]. In a series of 99 PMF patients, those with high mutation load (allele burden ≥ 50%) had significantly higher leukocyte count than those with a lower mutation load whereas no significant correlations were found between mutant allele burden and other hematologic variables, age, gender, spleen size and presence of splenomegaly [34].

In our study, we stratified our PMF patients into three different categories with regards to the JAK2V617F allele burden: JAK2V617F mutation-negative (n = 19), JAK2V617F-positive with low allele burden (allele burden ≤ 50%, n = 40) and high allele burden (allele burden > 50%, n = 18). We observed significant association between high mutation load and higher leukocyte counts, consistent with some previous reports [11, 12, 33, 34]. Confirming the observation of Guglielmelli et al, we found significant association between PMF patients with high allele burden and higher Hgb levels [33]. In addition, we observed higher Hct levels in PMF patients with high mutation load compared to the other two groups whereas no differences were found in age, platelet counts, LDH levels, presence of risk factors for cardiovascular diseases and the rate of phlebotomy. In our study, PMF patients with wild-type JAK2 had a higher rate of females than JAK2V617F-positive patients with low mutation load and high allele burden. Contrary to previous some reports but consistent with the study of Wang et al, JAK2V617F allele burden did not correlate with spleen size and prevalence of massive splenomegaly [11, 33, 34]. We observed no significant differences in the rate of splenectomy, rate of use of cytoreductive therapy and any other treatments in line with the observation of Tefferi et al [12]. Moreover, the prevalence of AHSCT did not differ in any of the different allele burden categories.

In our PMF patients, the prevalence of total thrombotic events, arterial thrombosis and venous thrombosis were similar between the groups, in agreement with the results of Tefferi et al [12]. The need for red blood cell transfusion in our PMF patients with low allele burden was higher than patients with wild-type mutational status and high allele burden. Our PMF patients with low mutation load displayed a higher yet not statistically significant rate of bleeding events compared to the other two groups.

Barosi et al reported that mutant allele burden in PMF did not significantly correlate with presence of abnormal cytogenetics [11]. Consistent with this observation, Guglielmelli et al reported that there was no statistically significant preferential clustering of unfavorable cytogenetic abnormalities in any of the different allele burden categories [33]. Distribution of karyotype categories was similar between our PMF categories in line with the above-mentioned studies [11, 33]. Wang et al found no significant correlation between JAK2V617F allele burden in PMF and IPSS or DIPSS risk category [34]. In another study, risk stratification according to the Dupriez prognostic scoring system did not differ between the groups [12]. But in our study, PMF patients with low allele burden showed a trend towards higher rate of DIPSS-plus high risk categories compared to PMF patients with high allele burden and wild-type mutational status.

Guglielmelli et al reported that PMF patients in the upper quartile had longer disease duration and the rate of mortality was higher in the lower quartile compared to those in upper quartiles [33]. Also, analysis of JAK2V617F-mutated patients after stratification according to the burden of JAK2V617F allele showed a significantly shorter survival in the lower quartile. On the other hand, rate of leukemic transformation did not differ in relation to JAK2V617F allele burden [33]. Barosi et al reported that PMF patients with high mutation load (allele burden > 50%) had longer disease duration and were more likely to experience leukemic transformation [11]. Tefferi et al reported that median follow-up, rates of death and leukemic transformation were similar in PMF patients with wild-type mutational status and different allele burden categories. In contrast, in the same study, Kaplan-Meier estimates revealed inferior OS and LFS in PMF patients with low JAK2V617F allele burden when compared to either higher allele burden or unmutated status groups [12]. Wang et al found no significant correlation between JAK2V617F allele burden and risk of blastic transformation [34]. In accordance with this study, JAK2V617F allele burden did not appear to have relevance in the evolution of leukemic transformation in patients with post-PV or post-ET myelofibrosis [35].

Durations of follow-up in our PMF patients were similar between the groups: mean 40.1 months (SD 33.9) in high mutation load, 42.8 months (SD 52.1) in low mutation load and 56.6 months (SD 48.7) in wild-type mutational status. We observed that PMF patients with low allele burden had a higher yet statistically insignificant rate of death compared to patients with high allele burden and wild-type mutational status. Similarly, the rate of leukemic transformation did not differ in any of the different allele burden categories.

In summary, we have documented that PMF patients with high mutation load (allele burden > 50%) had more pronounced myeloproliferative phenotype with higher leukocyte counts, Hgb and Hct levels, consistent with the study by Guglielmelli et al [33].

The design of this study might be questioned regarding that quantification of JAK2V617F allele burden was performed at variable times along the disease course because of the possibility of a time-dependent increase in mutation load. However, several studies reported that JAK2V617F mutation load in ET patients remain stable over many years [9, 10]. In addition, in the study by Campbell et al, none of the JAK2V617F-negative ET patients became positive after a median of 77 months [36]. In the study by Barosi et al, increasing allele burden was reported in PMF patients [11]. On the other hand, a substantial stability was observed in 88% (22/25) of patients with myelofibrosis while the increase in JAK2 expression in three of 44 patients (6.8%) did not correspond to disease progression [37]. As a whole, while a time-dependent increase in JAK2V617F allele burden has been reported in several cases, currently there is no longitudinal study with a consistent number of patients supporting the hypothesis that JAK2 mutational status varies significantly over time [38, 39].

In conclusion, our results point out that in ET patients, a high JAK2V617F allele burden is associated with more severe disease with lower Hgb and Hct levels, higher LDH levels, more prevalent massive splenomegaly, increased rate of DVT and higher prevalence of bleeding events. In PMF patients, our findings indicate that high JAK2V617F allele burden is consistent with a more pronounced myeloproliferative phenotype with higher leukocyte counts, Hgb and Hct levels. Finally, our data support the opinion that JAK2V617F allele burden correlates with clinical phenotype in Ph-negative MPNs.

Finally, somatic mutations of CALR (calreticulin gene) were detected in 20-25% of ET or PMF patients [40, 41]. Rumi et al reported that CALR-mutated PMF patients had a lower risk of developing anemia, thrombocytopenia, thrombosis and marked leukocytosis [42]. In another study, CALR-positive ET was associated with reduced leukocyte counts and Hgb levels and elevated platelet counts [43]. We aim to investigate the impact of CALR mutations in Ph-negative MPNs in our further studies.
